# Development of bespoke hardware and software to enable testing of a novel method of managing the charge and discharge of series-connected battery packs

**DOI:** 10.1016/j.mex.2023.102294

**Published:** 2023-07-20

**Authors:** John Hardy, John Steggall, Peter Hardy

**Affiliations:** a Intercal (UK) Ltd., 24 Hartlebury Close, Redditch B98 9LY, UK; b Indra Renewable Technology Ltd., Sparrowhawk Close, Malvern, Worcestershire WR14 1GL, UK

**Keywords:** Lithium-ion battery, Testing, Cell balancing, Test rig design

## Abstract

The novel feature of the method of battery management under development and testing is that routine balancing of the cells is eliminated throughout the service life of the battery pack. This requires preparation of the battery cells and configuration of the rigs in such a way as to ensure that the cells are accurately balanced on assembly and that thereafter there is no cell-to-cell variation in charge current or load at any time, measured to a low microamp level. In addition, the method requires a special charge control algorithm which was devised in order to accommodate cell-to-cell variations in capacity and dynamic response.

Comprehensive experimental testing of this method, which is fully described in the associated paper (Hardy et al., 2023), required the development of hardware and software which would combine the necessary functions of a battery test rig and a battery management system capable of carrying out the special method of charge control described below. These included:

• The automated control of contactors, loads and chargers to perform multiple charge/discharge cycles to predetermined patterns of current and maximum and minimum cell voltages.
• Monitoring of cell voltages, current and temperature and the provision of test and diagnostic data.
• Performing the safety functions of a Battery Management System to ensure that no cell was permitted to exceed limitations of current, voltage or temperature.

The hardware and software were developed through three phases of testing with the operational principles (but not all the hardware and software elements) carrying over from one phase to the next.

## Graphical abstract

**Figure:**
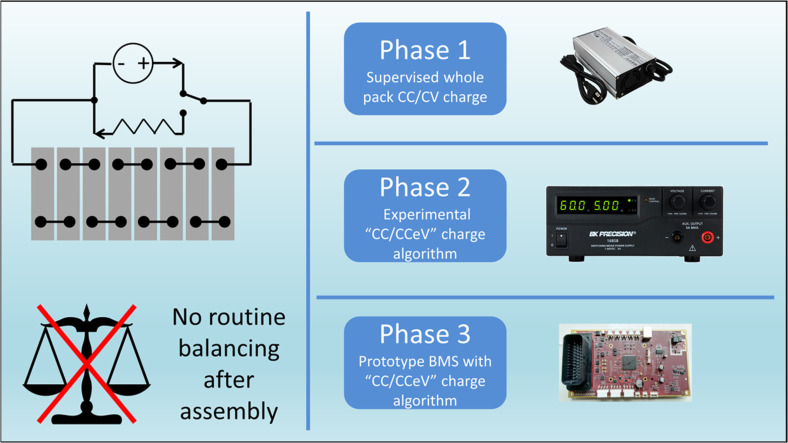

Specifications table

**Table:** 

Subject area:	Energy
More specific subject area:	Lithium-ion Battery Management
Name of your method:	Test rig design
Name and reference of original method:	N/A
Resource availability:	See Text

## Method details

### Background

The current state of the art in Battery Management Systems (BMS) for multi-cell lithium-ion batteries presumes that individual cells will inevitably drift apart in state of charge over time, unbalancing the battery. This is problematic in series-connected battery packs since discharge must be terminated when any cell reaches full discharge, and charge must be terminated when the first cell reaches full charge. The effect of loss of balance is thus to reduce the effective battery capacity.

Accordingly, virtually all BMSs include functionality to provide routine cell voltage balancing. This involves providing a separate connection to every cell, together with additional electronic components for each cell and appropriate control software. The most common design of balancing circuit process produces heat leading to potential issues with heat dissipation. Since a typical battery in an electric vehicle might have 100 cells connected in series, the wiring and control systems add weight and additional points of failure as well as significant cost to the battery installation.

The most insidious drawback of balancing circuits, however, is that routine balancing masks the emergence of cell faults, notably internal short circuits (ISC), which after initiation can evolve into sudden thermal runaway and fire. If routine balancing can be eliminated then an ISC developing in one of the cells will stand out clearly as the cell will lose charge compared to the other cells in the battery. This loss of charge is readily detected at an early stage of ISC evolution as a small but increasing drop in voltage compared with the other cells.

Ref. [1] reviews evidence related to cell behaviour that might cause unbalancing of a battery, and goes on to describe a series of 8 experiments or tests that show that:

• Routine cell balancing circuits can be effectively eliminated provided that certain precautions are taken in battery and BMS design, cell preparation and charging method, and that
• Elimination of routine cell balancing enables sensitive detection of voltage changes of the type caused by early stage ISCs, thus enabling timely detection well before the ISC evolves towards failure or thermal runaway.

This paper describes the design and operation of the test environments used in the tests described in [1]. The reader is referred to that paper for further detail of methodology and findings.

## Introduction

Hardware and software development continued through three phases of experimental testing as described in [1]. In summary this was:

Phase 1 – two tests using lithium iron phosphate cells, and off-the-shelf chargers applying a simple constant current/constant voltage (CC/CV) algorithm to the whole pack, under close supervision to prevent excess cell voltages.
Phase 2 – a five-test series using several other lithium-ion chemistries and introducing a novel approach to automated charging, “Constant Current/Constant Cell Voltage” (CC/CCeV), described below and in [1]). This is designed to optimize charge rates of the whole battery pack while preventing individual cell voltages from exceeding rated limits.
Phase 3 – a single test comparing the performance of a conventional Battery Management System (BMS) side-by-side with a reusable prototype BMS incorporating a more sophisticated embodiment of the CC/CCeV charging algorithm over 1000 charge-discharge cycles.

Accurate cell voltage balancing before assembly is a critical precondition for the method of battery management under investigation. The process used is described below.

### Phase 1

**Fig. 1:**
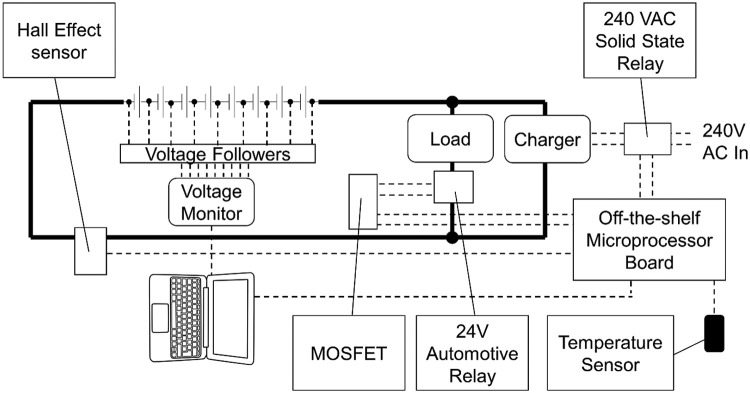
Phase 1 experimental testing - circuit diagram.

This early experimental testing, which was aimed simply at assessing voltage drift in the absence of routine cell voltage balancing, used commercial off-the-shelf smart chargers which applied a modified Constant current/Constant voltage (CC/CV) charge profile to the whole pack. A generic Arduino microcontroller board working with a PC was used to supervise cycling and record cell voltage, pack current and ambient temperature data in *.csv* files (Fig. 1). Although this simple charging method allowed some cells to peak above their target voltage, this was monitored to ensure individual cells voltage peaks were not excessively high. There may however have been some adverse effect on cycle life.

**Fig. 2:**
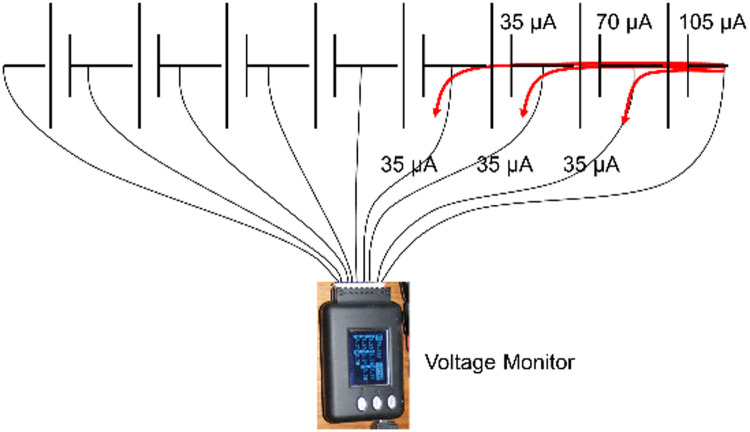
Unbalancing effect of cell voltage logger.

For the purposes of cell voltage monitoring, the initial intention had been to connect a low-cost “Junsi Cell-Log 8S” voltage logger unit, designed for transmitting cell voltages to a PC via USB. It was recognised, however, that this device would draw a current of approximately 35µA from the positive terminal of each cell. The unbalancing effect would have been cumulative (Fig. 2) and over all eight cells in the experimental pack the variation in net current drain would have amounted to at least 280 µA. This would have unbalanced the pack by about 0.2 Ah per calendar month.

**Fig. 3:**
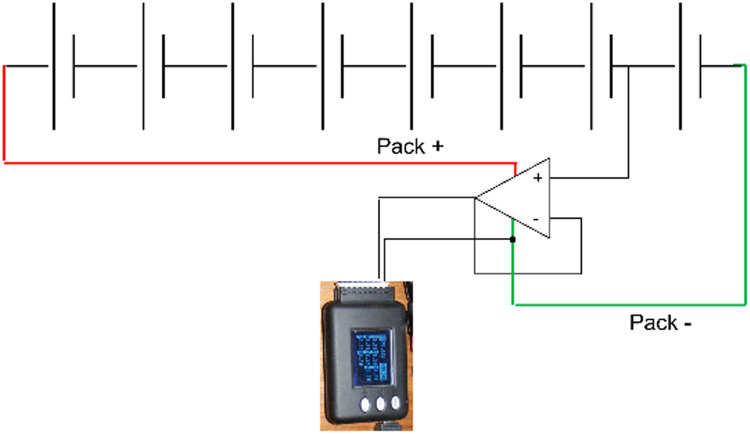
Operational Amplifier wired as voltage follower.

To overcome this, voltage followers based on operational amplifiers were placed between the logger and the cell terminals (Fig. 3). An operational amplifier wired in this way uses external power to replicate the input voltage at the output without drawing any current from the input.

Cycling was managed by switching the charger using a solid-state relay (SSR) in the mains supply to the charger.

**Fig. 4:**
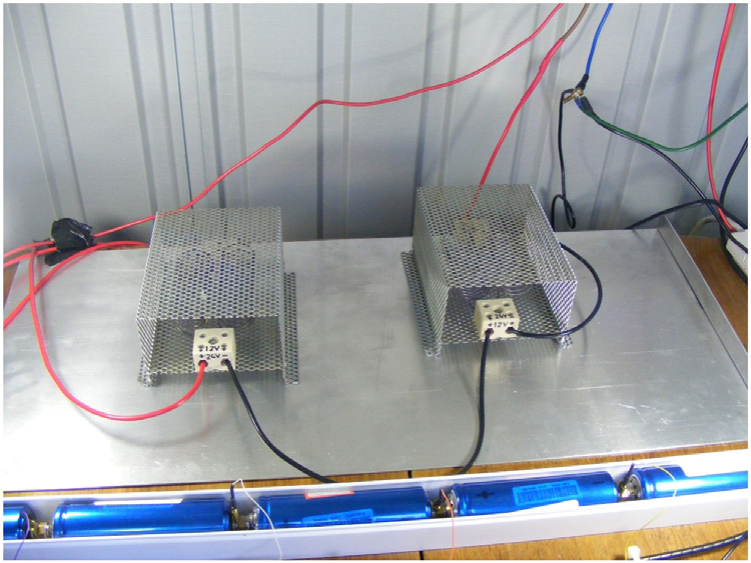
Phase 1 Resistive load.

The load was resistive (Fig. 4), switched using a simple 24 V automotive relay which was in turn switched using a MOSFET.

Current was measured using a Hall effect sensor. The Arduino microcontroller board was programmed using its own scripting language and communicated with the PC which was running code written in the Processing scripting language.

The equipment used for Phase 1 is summarised in Table 1.

**Table 1:** Phase 1 Equipment summary.

Controller	Cell Voltage Monitoring	Charger	Current Measurement	Load	Temperature Monitoring
*Arduino Mega* off-the-shelf microcontroller board + PC	*Junsi Cell* Log *8S* plus bespoke operational amplifier board	Unbranded LiFePO4 29.2 V smart charger	*LEM LTSR 15-NP* Hall Effect Sensor	Resistive	*Maxim DS18B20*

### Phase 2

**Fig. 5:**
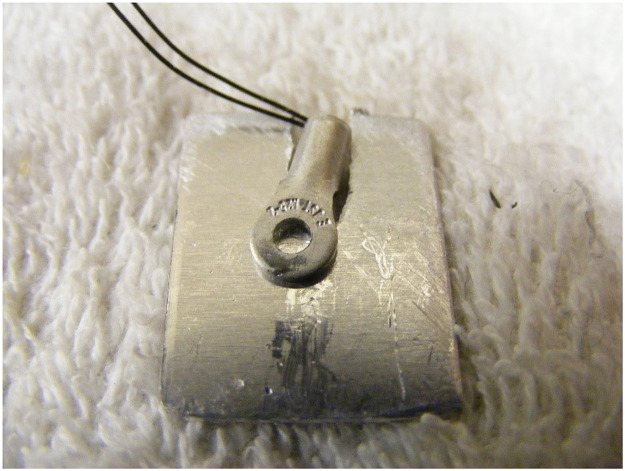
Phase 2 Thermistor used to monitor terminal temperatures.

The test rigs for Phase 2 were refined, principally in order to apply the experimental CC/CCeV charging protocol This method, described in more detail below, modulates whole pack charge current to the maximum which at any time can be accommodated without causing any cell to go over voltage. The rigs also provided for an additional parameter, cell terminal temperature, to be monitored. The same combination of Arduino and PC was used, the load was resistive as before and the current measured using a Hall effect sensor as in Phase 1. The refinements over Phase 1 were:

• A variable voltage laboratory power supply was used instead of a smart charger. This allowed for the fine control of charging current required to implement the experimental CC/CCeV charging protocol.
• The Arduino was programmed to control the Power Supply, setting current and voltage to implement the charging protocol.
• Cell positive and negative terminal temperatures were measured using thermistors (Fig. 5).

Use of a Kunkin KL284A load was considered but it was concluded that this offered little advantage over the simpler resistive load since it could only be set manually.

The equipment used for Phase 2 is summarised in Table 2.

**Table 2:** Phase 2 Equipment summary.

Controller	Cell Voltage Monitoring	Charger	Current Measurement	Load	Ambient Temperature	Cell Temperatures
*Arduino Mega* off-the-shelf microcontroller board + PC	*Junsi Cell* Log *8S* plus bespoke operational amplifier board	*BK Precision 1687B* Power Supply	*ACS712* Hall Effect Sensor	Resistive	*Maxim DS18B20*	*NTCALUG03A103HC* or similar thermistors read by 12-bit TI *ADS1015* or 16-bit *ADS1115* ADCs with I2C interface

Designing most of the required software was reasonably straightforward. The CC/CCeV algorithm provides that on charge during each loop of the software the voltage of the cell is checked and if the highest cell voltage exceeds a given target value, the current is decremented by a fixed value, which was determined by experimentation. Although this worked well for this cycle testing where charging always started from a discharged pack, it was recognised that a real-world BMS would need a more sophisticated implementation of the algorithm. This was developed for the prototype BMS used in Phase 3, as described below.

### Phase 3

**Fig. 6:**
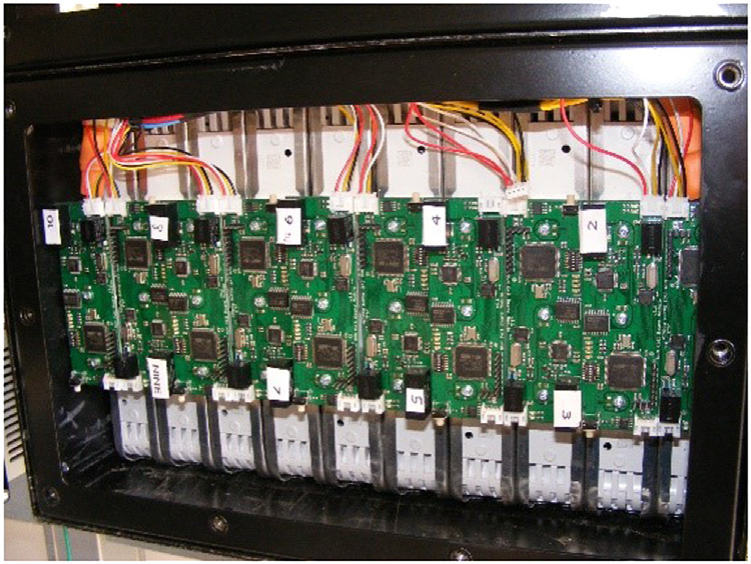
Cell boards in Prototype battery pack for a Quad bike.

**Fig. 7:**
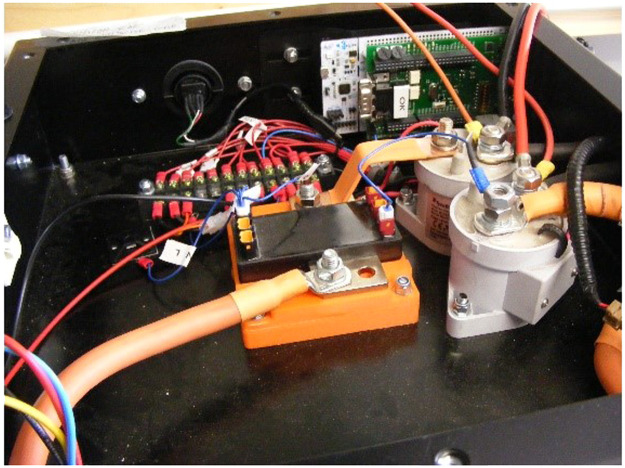
Smart Shunt (centre foreground), twin contactors (right foreground) and Nucleo Board (background) in the Quad bike pack.

The Phase 3 experiment was conducted some time after Phases 1 and 2, during which period development had proceeded towards a reusable prototype BMS incorporating the operating principles developed and tested during Phases 1 and 2. An early embodiment of this was incorporated in a battery pack configured to simulate an airliner Auxiliary Power Unit battery. A more sophisticated version was used first in a converted electric Quad using Nissan cells. This latter version used cell boards to read cell voltages and terminal temperatures, and to broadcast them on CAN. A central master unit consisted of an STM32 Nucleo board with third party programming to control contactors, together with a Zivan SG3 charger, implementing the CC/CCeV protocol. Current and pack voltage was read using an Isabellenhutte CAN-enabled smart shunt (Figs. 6 and 7).

**Fig. 8:**
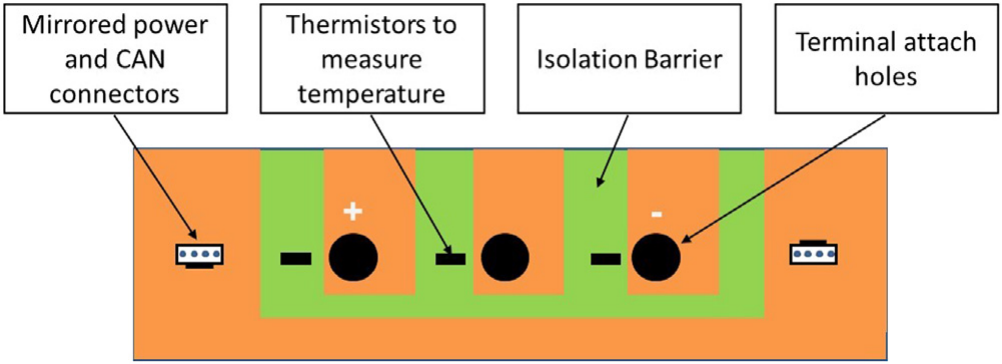
Schematic of cell board isolation.

The experience gained in this development work was used to specify a further design iteration providing a complete reusable prototype for use in the Phase 3 experiment. This was implemented in bespoke hardware and with software designed and programmed using STM32 microcontrollers. The overall architecture was the same as developed for the Quad bike, comprising a Master and Cell Boards connected via Controller Area Network (CAN) with a smart shunt to measure current. The function of the Cell Boards was to transmit cell voltage and terminal temperatures to the Master on a CAN bus. The electronics were isolated from cell voltage so that there were no high voltages outside the Cell Board (Fig. 8).

The Cell Boards were powered externally, not by the cell itself, in order to eliminate any risk of unbalancing loads resulting from running the electronics.

The Cell Boards were connected to the Master using a simple four-wire daisy chain (12v+/12V- to power the board electronics plus CAN High and CAN low for cell voltage and terminal temperature data). In a high voltage pack this arrangement is much simpler than the wiring required for a conventional centralised BMS which incorporates a separate connection to each cell used for cell voltage monitoring and balancing.

**Fig. 9:**
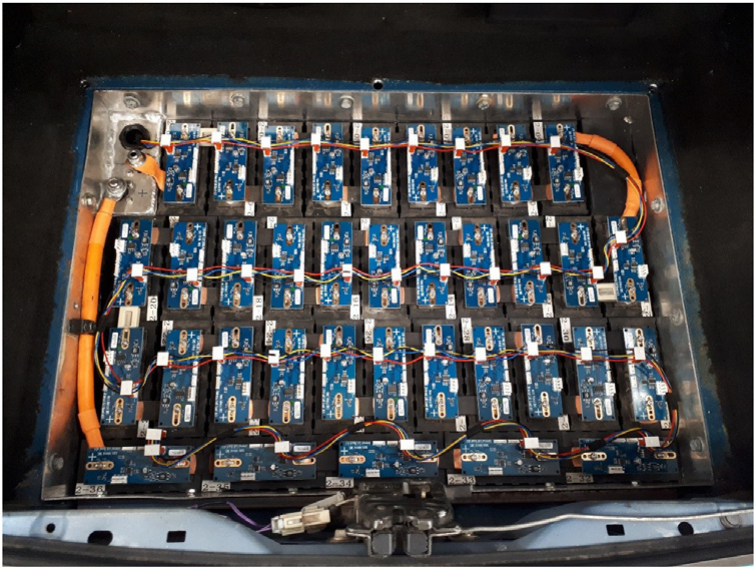
Cell Board installation in a test bed vehicle. The four-wire daisy-chain is power (positive and negative) and CAN (high and low).

Eliminating these connections not only reduces cost and complexity, it also improves safety during maintenance by reducing high voltage wiring in the BMS. Fire risk is also reduced by the absence of opportunities for chafing of high voltage wires (Fig. 9).

The Cell Board CAN bus terminates at the Master which accumulates the cell voltages and terminal temperatures. The Master also takes current and total pack voltage data over CAN from the smart shunt, similar to the one in Fig. 7. It controls the pack contactor(s) and implements several fail-safe mechanisms to issue warnings, invoke limp mode or shut the pack down in the event of fault conditions such as a missing cell board, a mis-match between pack voltage and total cell board voltages, excessive current, low cell voltages or terminal temperatures outside limits.

**Fig. 10:**
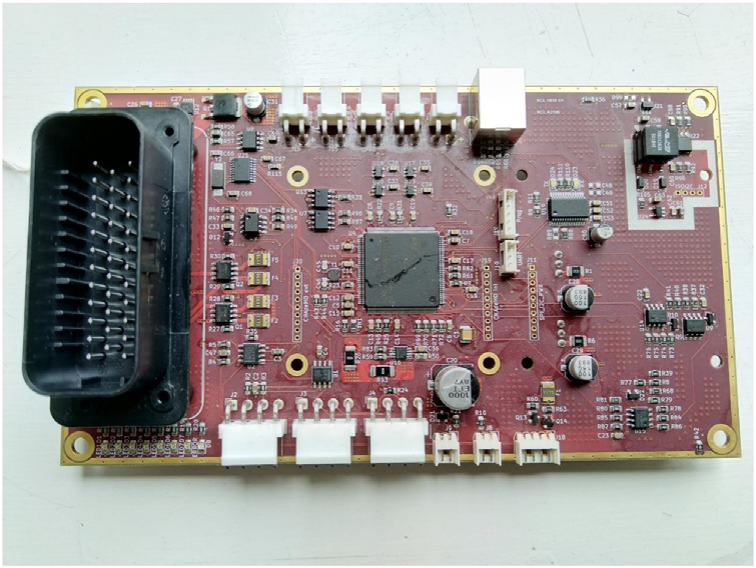
The Master board used in Phase 3.

The Master (Fig. 10) also controls the charging process, using a more sophisticated implementation of the CC/CCEV algorithm than was applied in the Phase 2 experiments, and outputs data on an external CAN bus.

The development of this hardware and software provided high precision charge control and more accurate data logging at shorter intervals. It also demonstrated the practicality of producing a fully functioning BMS applying the operating principles described in [1].

**Fig. 11:**
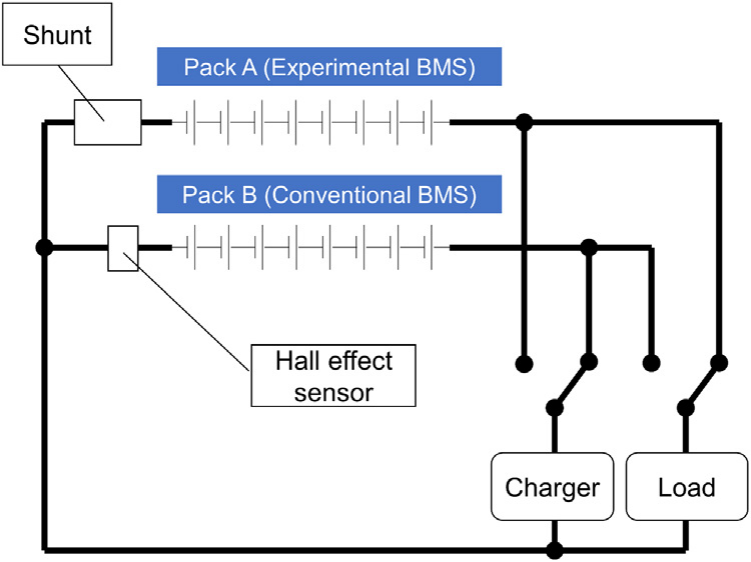
Phase 3 Test rig wiring diagram (Fuses, plus control and data collection wiring, not shown).

As described in detail at [1] the test rig for the Phase 3 experiment was designed to enable comparative performance monitoring of two separate battery packs, one managed with the equipment described above, and the other using a commercial “Orion 2″ balancing BMS manufactured by Ewert Energy Systems. A single electronic load and a single electronic power supply were used for both packs, with cycles interleaved so that one pack was charging whilst the other was discharging (Fig. 11).

It was essential to avoid the risk of occasional shut down mid-cycle that may occur using Windows PC's, for example when installing an operating system update. For most of Phase 3, therefore, a mini-PC running Linux was used in “headless” mode, without screen or keyboard. This was accessed via a Virtual Network Computing (VNC) graphical desktop-sharing app from a remote PC. A small Uninterruptible Power Supply (UPS) was used to avoid the risk of power outages.

**Fig. 12:**
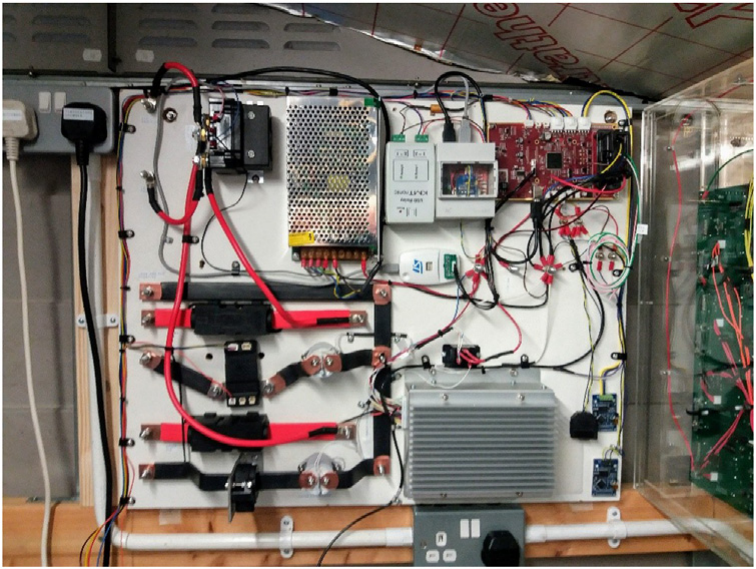
The final Phase 3 test rig. The Pack A experimental BMS master is top right. The Pack B conventional BMS is the finned aluminium box bottom centre-right.

The complete rig included a 12 V power supply for the electronics, high current fuses, bus bars and contactors (Fig. 12). As well as scheduling charge and discharge, the Linux mini-PC was programmed to swap changeover relays and write data output by both BMSs to file at 1 s intervals.

## Results

Table 3 lists the cell types and other test metadata The results and analysis for all three phases are detailed in [1], and are not therefore repeated here.

**Table 3:** Test metadata.

Phase	Test Number	Cell Type	Cycles	Total CSV files (Mb)	Sampling interval (seconds)
Phase1	1	Headway 38120S	575*	11.3	30
	2	CALB CA40fi	2008	104	30
Phase 2	3	GP1865L220	159*	12	30
	4	GP1865L220	659	28.2	30
	5	Panasonic NCR18650A	319*	22.4	30
	6	Samsung ICR18650–22P	638	482	30
	7	Samsung ICR18650–22P	719	45.2	30
Phase 3	8	Nissan Gen 4	1004	10,400	1

⁎ A cell failure caused early termination of the test.

**Fig. 13:**
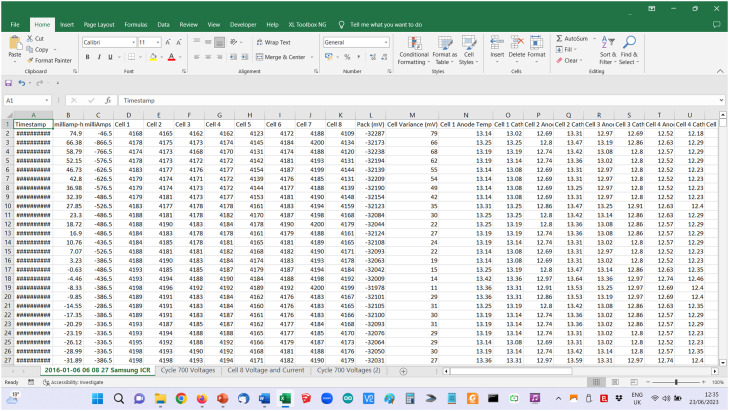
Sample CSV data.

The raw data was collected in CSV (comma separated) files. Note that the file size depends on the sampling interval, the number of columns of data collected, the charge and discharge currents, length of rest periods, battery capacity and test duration. An example of the raw data collected forms Fig. 13.

**Fig. 14:**
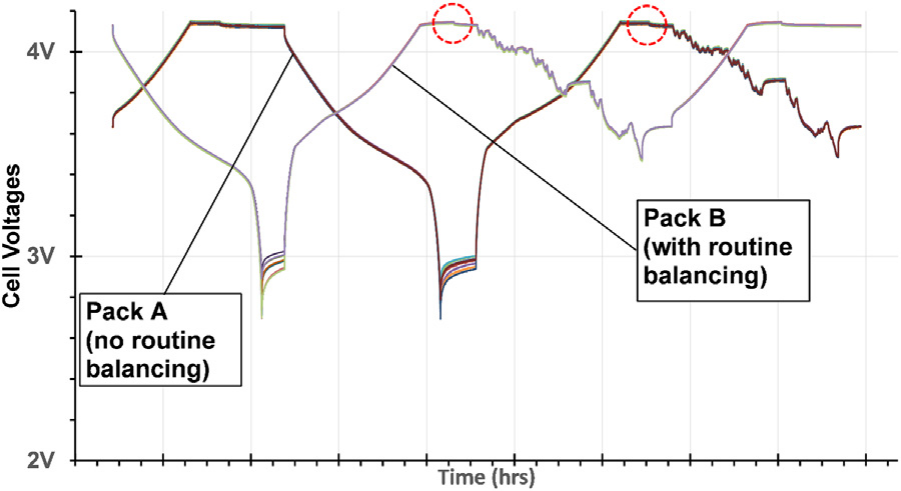
Sample of data plotted and annotated in Excel.

Data were plotted using Microsoft Excel charts and annotated as required (Fig. 14).

Note that Figure 14 illustrates a test that had two battery packs – one fitted with a conventional balancing BMS and one with the prototype non-balancing BMS

### Data validation

Cell voltages were cross checked with a Volt-Ohm metre and currents with an in-line or clamp ammeter.

In Test 8, a precision power supply and load were used, and both returned pack voltage and pack current. These values could be compared with the equivalent values returned by the two BMSs in order to verify that the latter were correct.

In addition, the prototype BMS measured pack voltage independently of cell voltages so the total of all cell voltages could be compared with the reported pack voltage.

### Bottom balancing

In the absence of routine cell voltage balancing (apart from the conventional “Pack B” BMS used in Phase 3) pack performance depended on accurate balancing prior to assembly. Cells were “bottom balanced” (i.e. near full discharge) since at this point the higher rate of change of voltage against state of charge allowed greater precision.

**Fig. 15:**
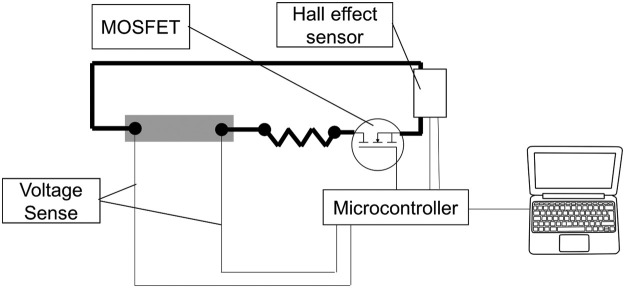
Bottom balancer schematic.

Since lithium-ion cells exhibit voltage recovery following cessation of current flow, with a voltage drop after charging ceases and voltage rise after discharge, balancing needs to be carried out with care. The approach used was to connect each cell to a balancing rig as shown schematically in Fig. 15. A microcontroller was configured to allow discharge through a resistor until a pre-determined voltage was reached, at which point it would switch the MOSFET off and on (initially very rapidly) to maintain the selected voltage. A PC was used to record data for analysis and trouble-shooting.

Several considerations apply to this process:

• It is critical that the voltage sensing lines are independent of the main circuit: the effect otherwise would be to distort the voltage readings due to voltage drop in the main circuit.
• Assuming that the cells start out at a similar state of charge, it was found that a balancing period of 15 min at the selected voltage was normally sufficient.
• The charge history of the cells is important. If the cells in a pack had previously been held at significantly varying states of charge, the so-called “anode overhang” effect described in [1] could induce cell voltage drift, continuing over a prolonged period.

## Conclusion

Testing during Phase 3 involved running two identical packs, one fitted with a conventional balancing BMS and one with the prototype non-balancing BMS, to around 1000 deep cycles over a range of conditions during which the rig described here functioned reliably and effectively, allowing precise control over charging together with precision data recording covering all the key parameters. The rig specification applied for Phase 3 thus appears suitable for further testing and as a basis for full prototype development.

## Ethics statements

N/A

## CRediT authorship contribution statement

**John Hardy:** Conceptualization, Methodology, Validation, Formal analysis, Writing – original draft, Visualization. **John Steggall:** Software, Resources, Data curation. **Peter Hardy:** Writing – review & editing, Supervision, Project administration, Funding acquisition, Supervision.

## Declaration of Competing Interest

The authors declare the following financial interests/personal relationships which may be considered as potential competing interests:

John Hardy and Peter Hardy are directors of Intercal. Intercal holds patents in related areas.

## Data Availability

Data will be made available on request.
